# Determinants of vaccination dropout among children 12-23 months age in north Gondar zone, northwest Ethiopia, 2019

**DOI:** 10.1371/journal.pone.0246018

**Published:** 2021-02-08

**Authors:** Muluken Genetu Chanie, Gojjam Eshetie Ewunetie, Asnakew Molla, Amare Muche

**Affiliations:** 1 Department of Health Systems Planning and Management, School of Public Health, College of Medicine and Health Sciences, Wollo University, Dessie, Ethiopia; 2 Department of Medical Laboratory Sciences, Denbya Primary Hospital, North Gondar, Gondar, Ethiopia; University of Mississippi Medical Center, UNITED STATES

## Abstract

**Background:**

Vaccination is a proven tool in preventing and eradicating childhood infectious diseases. Each year, vaccination averts an estimated 2–3 million deaths from vaccine preventable diseases. Even though immunization coverage is increasing globally, many children in developing countries still dropout vaccination. The objective of this study was to identify determinants of vaccination dropout among children age 12–23 months in North Gondar, North west Ethiopia.

**Methods:**

Community based unmatched case-control study was conducted in north Gondar from March 1–27, 2019 among 366 children age 12–23 months (92 cases and 274 controls). Multistage sampling was used for reaching to the community. Data were collected from mothers who had 12–23 months age children using a pretested structured face to face interview. Data were entered using Epi info v. 7 and exported to SPSS v. 20 for analysis. On multivariable logistic regression variables with P-value <0.05 at 95% CI were considered statistically significant.

**Result:**

Counseling for mothers about vaccination (AOR = 7.2, 95% CI: (2.93–17.5)); fear of vaccine side effects (AOR = 3.5, 95% CI: (1.56–8.12)); PNC attended (AOR = 3.6, 95% CI: (1.52–8.39)) and mothers not received tetanus toxoid vaccination (AOR = 2.4, 95% CI: (1.03–5.35)) were found risk factors of vaccination dropout.

**Conclusion:**

Counseling on vaccination, fear of vaccine side effects, PNC attended and mothers’ tetanus toxoid vaccination status during ANC visit were found risk factors. Management bodies and health workers need to consider “reaching every community” approach, Counsel every mother at any opportunity, and provide TT vaccination for all pregnant mothers helps to reduce vaccination dropout among children.

## 1. Background

Childhood vaccination is one of the most cost-effective public health interventions to reduce child morbidity and mortality. Besides attainment of high coverage with potent vaccines, receiving child full course of recommended immunization doses administered at the appropriate age is essential to reduce the incidence of vaccine-preventable diseases in children. So assuring that children receive all doses of all vaccines before their first birthday is necessary for childhood vaccination status [1, 2]. According to guidelines developed by the World Health Organization (WHO), children are considered as fully vaccinated when they have received a vaccination against tuberculosis Bacillus Chalmette Guerin (BCG), three doses of Pentavalent vaccine Diphtheria, pertussis and Tetanus-Hepatitis B-Hemophilus influenza type b (DPT-Hep B-Hib), pneumococcal conjugated vaccine (PCV) and polio vaccines, two dose of Rota virus, and a measles vaccination by the age of 12 months. Considering this incomplete vaccination can be defined children who missed at least one dose of the ten vaccines before 12 months [3, 4].

Center for disease control (CDC) and the World Health Organization (WHO) evaluated the WHO and United Nations Children’s Fund (UNICEF) global vaccination coverage estimates to describe changes in global and regional coverage. As of 2016 global routine vaccination coverage, DTP3 coverage ranged from 74% in the African Region to 97% in the Western Pacific region [5].

Dropout rate is the rate difference between the first and the last dose or the rate difference between the initial vaccine and the last vaccine. It is an indicator of immunization program performance and estimated to be 5% in 2016 for the 3-dose DTP series, with dropout highest in the African Region (11%) and lowest in the Western Pacific Region (0.4%). In routine expanded Program on immunization (EPI) programs, drop ‐out rate higher than 5% usually indicates quality problem with the program and need to be addressed [5]. Dropout rate is used to measure program continuity and follow up. The dropout between the first and third doses of DPT‐HepB‐Hib, in particular is the best indicator as this vaccine is not typically given during campaigns [6].To achieve maximal protection against vaccine-preventable diseases, a child should receive all vaccines within recommended intervals [7].

In Ethiopia, vaccination is being given on routine and outreach basis. The routine vaccination schedule recommends that infants should be vaccinated starting from birth and complete their vaccination before 1 year of life with one dose bacillus Calmette Guerin (BCG) at birth or as soon as possible and oral polio vaccine initial doze (OPV0). Three doses of OPV, Pentavalent, PCV, Rota1, Rota2 and are given at interval of 4 weeks duration at the 6th, 10th and 14th weeks, respectively and finally measles vaccine is given at the age of 9 months. A child is said to be fully vaccinated if he/she received all vaccines according to the schedule [8].

The 2016 Global routine immunization coverage report showed that among approximately 20 million children who did not complete the 3-doses of DPT series, 12.9 million (66%) did not receive any DPT doses, a decrease from 79.4 million in 1980, and 6.6 million (34%) started, but dropped out and did not complete the DTP series. The largest proportions of infants who drop out from vaccination were Eastern Mediterranean (15%) and African countries (17%). National DPT1 to DPT3 dropout rates was lowest in the Western Pacific Regions (0.4%) but highest in the African Regions (11%). Among the 19.5 million children worldwide who did not received DPT3 doses during the first year of life, 11.8 million of them (61%) were lived in 10 countries: India (16%), Pakistan (7%), Indonesia (6%), Iraq (3%), Angola (2%), Brazil (1%), and South Africa (1%) [5], the Democratic Republic of the Congo (3%), Nigeria (18%), Ethiopia (4%).

A report from WHO revealed that around 60% of children’s who were not reached with routine immunization services were from 10 countries where majority are from sub-Saharan African countries [9]. And five of those African regions including Ethiopia were the region that continues to even increase further the pool of unimmunized children [10].

The Ethiopian Demographic Health Survey (EDHS) 2016 report revealed that close to two in every five children aged 12–23 months (39%) received all basic vaccinations at some time, and 22% were vaccinated by the appropriate age. It was found that the percentage of children aged 12–23 months who are fully vaccinated increased from 24% in 2011 to 39% in 2016 [11].

Due to its large size population, the country still has a large number of unvaccinated children and there are huge variations in immunization coverage among regions. Due to the topographic and climatic situation as well as limited capacity of the EPI programs [12].

Study conducted in Ethiopia showed that 66% of the children received BCG vaccine and 56% received measles vaccine. A relatively higher percentage of children received the first dose of DPT (64%). However, only 37% received the third dose of DPT, reflecting a dropout rate of 42%. More than eight out of every ten children (82%) received the first dose of polio, but only about four in ten (44%) received the third dose, reflecting a dropout rate of 46%in Ethiopia [13].

According to the Ethiopian Demographic and Health Surveys (EDHS) 2016 report, there were factors that contribute for vaccination dropout in children. About 3 in 10 (31%) of children whose mothers have no education are fully vaccinated compared with more than 7 in 10 (72%) of children whose mothers have more than a secondary education. Similar patterns are observed by household wealth. The survey also indicated that there was a wide variation and discrepancies among regions regarding full immunization coverage ranging from 89% in Addis Ababa to 15% in Afar region [14]. Therefore, the aim of this study was to identify determinants of vaccination dropout among children age 12–23 months in North Gondar.

## 2. Materials and methods

### 2.1. Study design and setting

Community based unmatched case control study design was conducted in North Gondar from March 1-27/2019, which is constituted from 24 districts. It is located 190Km from the city of Amhara region, Bahirdar and 727 km from Addis Ababa. There were 234,353 reproductive age women, 12,160 under five children and 2,869 infants in north Gondar. There were 84 health centers and 264 health posts all deliver static and out rich vaccination services within this zone.

### 2.2. Population

All children of age 12–23 months in North Gondar during the study period.

#### Cases

Were children in the age group of 12–23 months who did not complete their vaccination before celebrating his/her first birthday in the study area.

#### Controls

Children who were in the age group of 12–23 months and completed their vaccination during the data collection period.

### 2.3. Sample size determination and sampling procedures

Sample size was determined using Epi-info version 7.0.8.3 and based on the following assumptions: Precision 5% at 95% confidence level, power of 80%. The ratio of controls to cases (r) = 3, OR = 2.549 [15]. Considering the design effect of 1.5, and possible non-response rate 10%, a sample size of 366 (92- cases and 274- controls) were included in the study. The study participants were drawn using a multi-stage sampling technique. First six districts (from 24 districts) and then 18 kebeles (from 184 kebeles) were selected by lottery method in North Gondar zone taking into consideration the feasibility of the study. In each selected kebeles, a list of cases, controls and their full addresses were got from the list of records of nearby health centers and health posts that provide child health services, including EPI. Then sampling frame was prepared from each selected kebeles and sorted out in consecutive order. Finally, systematic sampling technique was applied to access cases and controls from the sampling frame of both cases and controls for data collection. In all 18 kebeles the total number of children in the age group 12 to 23 months were 2,187. To create equal chance in the selection of children, proportional allocation technique for both cases and controls were performed across each selected kebeles. Based on proportional allocation, the 366 (92 cases and 274 controls) study participants were distributed to each kebeles accordingly. An interval of K was calculated and then cases and controls were accessed accordingly. Mothers/care givers of the selected children were traced to participate in the study and vaccination dropout of children were confirmed by mothers/care givers recall (self-report) by reviewing child immunization cards and record review from the respective health facilities during data collection.

### 2.4. Inclusion and exclusion criteria for cases and controls

All children age 12–23 from all selected kebeles were included in the study. But critically ill mothers/caregivers of those children and those who lived less than six months in the kebeles were excluded from the study as it was difficult to access full data.

### 2.5. Study variables

Vaccination dropout (Yes/No) was used as a dependent variable; whereas the independent variables were Maternal socio-demographic factors like maternal age, educational status,employment status,family size; Maternal knowledge about vaccination, life style and health care utilization: Place of delivery, ANC follow up, PNC service utilization, parity, forgetting appointment, knowledge about vaccination, Mothers/care takers way of life, mother/caretaker too busy; Institutional factors: distance from health facility, absence of providers, Vaccination stock out, HW/HEW home visit, waiting time/vaccination day postpone, Counseling on vaccination completion; Child factors: child sickness, sex of child, age of child, fear of side effect.

### 2.6. Operational definitions

#### Vaccination dropout

It was used for the vaccinations which have consecutive doses and was measured by asking yes/no questions (In Ethiopia it is Pentavalent vaccines 1–3 doses).

#### Full vaccination

In this study it was a situation whereby the child took the entire recommended vaccine doses by one year of age;

#### Vaccination dropout rate

In this study it is the rate difference between the first and the last dose or the rate difference between the initial vaccine and the last vaccine;

#### Defaulter

In this study it was a situation occurs when the child missed at least one of the recommended vaccine doses;

#### Good Knowledge

Mothers who correctly answer 60% and more of the knowledge related questions were considered as having good knowledge [16].

### 2.7. Data collection tools and procedures

Data collection tools were adopted from reviewing different literatures [13, 17, 18] and prepared accordingly. Data were collected using a pre-tested, structured and face to face interview questionnaires. The data collection tools were first prepared in English and then translated to Amharic. The second version of the questioner was translated into the original one to check its consistency. Six diploma Nurses for data collection and two BSc graduate supervisors were recruited to check daily for the completeness, clarity and consistency of the questionnaires and to give appropriate supportive supervision during the data collection process on the field.

### 2.8. Data quality management

Pre-test was done on 37(10%) of the total sample size one week prior to data collection other than the selected kebeles (Maksegnit district). One day training was given for data collectors and supervisors that focused on understanding of the research questions, sampling techniques, data handling, ethical conduct, and quality of data collection. The supervisors and the principal investigator were making frequent checks on the data collection process to ensure the completeness and consistency of the collected data. During data processing the data were also be cleaned, coded and entered properly.

### 2.9. Data processing and analysis

The data were checked for completeness, missing value and inconsistencies before data entry. The collected data were coded and entered in Epi-info v. 7.0.8.3 and exported to SPSS v. 20. Descriptive statistics like mean, standard deviation and percentages were calculated. Binary logistic regression analysis was conducted primarily to check which variables have association with the dependent variable individually. Variables which had association with the dependent variables at P-value ≤ 0.25 [19] were fitted to multivariable logistic regression model. The technique used was backward stepwise method. Adjusted odds ratio with 95% confidence level were computed for both cases and controls to show the strength of association between dependent and independent variables. In the multivariable logistic regression analysis variables having p-value < 0.05 were used to declare as significant predictors of vaccination dropout at 95% CI.

### 2.10. Ethical considerations

Ethical clearance was taken from ethical review board of University of Gondar, College of Medicine and Health Sciences. Letter of permission to conduct the study was obtained from administrative office of each kebeles’ office. Written informed consent was provided for participants before data collection. They were informed that participating in the study was voluntarily. The right to withdraw from the study at any moment during the interview was assured. No personal identifiers were used on data collection form. The recorded data was not accessed by a third person except the principal investigator, and was kept confidentially and anonymously. The ethical consideration of this study strictly followed the Helsinki Declaration.

## 3. Result

### 3.1. Socio-demographic factors

A total of 90 cases and 269 controls (with 98% response rate) were participated in the study. The mean age of mothers and children were 28.37 (SD±5.89) years, and 16.92 (SD±3.172) months, respectively. Majority of cases 46 (46.3%) and of controls 152 (56.4%) were males. Most respondents of cases 75(83.3%) and controls 213(79.1%) were Amhara by ethnicity; 68(75.9%) of cases and 210 (77.9%) of controls were Christian by religion, and 65 (72.2%) of cases and 249 (92.6%) of controls were married. Majority of the mothers of both 68 (75.9%) cases and 142 (52.8%) controls had no formal education, and 75 (83.3%) of cases and 211 (78.5%) of controls were housewives (Table 1).

**Table 1:** Socio-demographic factors of mother/care givers on determinants of vaccination dropout among children age 12–23 months in North Gondar, Northwest Ethiopia, 2019 (n = 359).

Variables	Category	Vaccination status	
		Cases	Controls
Age of mothers	15–24	25(27.8%)	78(28.8%)
	25–34	42(46.3%)	155(57.7%
	35–44	23(25.9%)	36(13.5%)
Religion	Orthodox	68(75.9%)	210(77.9%)
	Muslims	22(24.1%)	59(22.1%)
Marital status	Married	65(72.2%)	249(92.6%)
	Widowed	15(16.7%)	10(3.7%)
	Divorced	10(11.1%)	10(3.7%)
Educational level	No formal education	68(75.9%)	142(52.8%)
	Primary school (1–8)	22(24.1%)	127(47.2%)
Ethnicity	Amara	75(83.3%)	213(79.2%)
	Tigray	15(16.7%)	56(20.8%)
Occupation	House wife	61(68.3%)	211(78.5%)
	Employee (governmental)	14(15%)	38(14.1%)
	Daily labor worker	10(11.1%)	10(3.7%)
	Merchant	5(5.6%)	10(3.7%)

### 3.2. Maternal health care utilization related factors

Health service related characteristics of respondents showed that most cases 74 (82.3%) and controls 246 (91.5%) care givers of their children were their mothers. Forty six (51.1%) of cases and 158 (58.9%) controls had followed antenatal care follow up and less than half of controls 107 (39.9%) attended fourth antenatal care visit and 23 (25.9%) of cases followed antenatal care at least one time during their pregnancy. Majority of cases 58 (64.8%) and around half of controls 132(49.1%) had not received tetanus toxoid vaccination during antenatal care. Regarding delivery place, majority of cases 73 (81.5%) and controls 167 (62%) delivered at home. More than half of cases 48 (53.7%) and 101 (37.4%) of controls had not attended postnatal care services. In addition to these, majority of care givers among 63(70.4%) of cases and 206 (76.7%) of controls were not busy to vaccinate their children (Table 2).

**Table 2:** Health care utilization of mothers/care givers on determinants of vaccination dropout among children age 12–23 months in north Gondar, Ethiopia, 2019 (n = 359).

Variables	Category	Vaccination status	
		Cases	Controls
Primary Care givers	Mother	74 (82.3%)	246 (91.5%)
	others	16 (17.7%)	23 (8.5%)
Parity	1–3	35(38.9%)	175(65%)
	4–6	28(31.5%)	76(28.2%)
	7–12	27(29.6%)	18(6.7%)
ANC attendance	Yes	47(51.9%)	158(58.9%)
	No	43(48.1%	111(41.1%)
How many times ANC attended	One times	21(10.2%)	16(7.8%)
	Two times	15(7.3%)	33(16.3%)
	Three times	11(5.3%)	41(20%)
	Four times	7(3.4%)	61(29.7%)
TT vaccine received	Yes	32(35.2%)	132(50.9%%)
	No	68(64.8%)	127(49.1%)
How many times TT received	One time	15(16.7%)	58(21.5%)
	≥ 2 times	23(26%)	92 (34.4%)
	None	52(57.3%)	19 (7.4%)
Place of delivery	At home	73(81.5%)	167(62%)
	Health institutions	17(18.5%)	102(38%)
PNC attended	Yes	42(46.3%)	168 (62.6%)
	No	48(53.7%)	101(37.4%)
Busy to vaccinate child	Yes	27(29.6%)	63(23.3%)
	No	63(70.4%)	206(76.7%)

### 3.3. Maternal knowledge about vaccination of children

Majority of caregivers of cases 75(83.3%) and controls 259(96.3%) had heard about vaccination. Major sources of information for caregivers of cases and controls were health workers for 28(31.5%) and 82(30.1%) respectively. Majority of Mothers/care givers of cases 67(74.1%) and controls 249(92.6%) knew immunization was considered to be important to prevent disease while mothers/care givers of cases 15(16.7%) and controls 15(5.5%) believed it helped for child health.

Respondents knowledge about the age of child to begin vaccination showed that 30 (33.3%) of cases and 173 (64.4%) of controls said just after birth; and 15(16.7%) of cases and 84(31.3%) of controls responded 6 weeks after birth. On other hand, 35(38.9%) of cases and 7(2.5%) of controls don’t knew the age on which children begin vaccination. Among controls 132(49.1%) knew four sessions are needed to fully complete child vaccination (Table 3).

**Table 3:** Knowledge of mothers/care givers on determinants of vaccination dropout among children age 12–23 months in North Gondar, Ethiopia, 2019 (n = 359).

Variables	Category	Vaccination status	
		Cases	Controls
Heard about vaccination	Yes	75(83.3%)	259(96.3%)
	No	15(16.7%)	10 (3.7%)
From where heard about vaccination	Health workers at health facility	28(31.5%)	81 (30.1%)
	health extension worker	48(53.3)	160 (59.5%)
	TV	7(7.4%)	8(2.8%)
	Others	7(7.9%)	19(7%)
Benefit of vaccination	To prevent diseases	66(74.1%)	249(92.6%)
	For child health	6 (6.7%)	15(5.5%)
	Others	8(9.3%)	5 (1.8%)
Age for beginning vaccination	at birth	30(33.3%)	173(64.4%)
	Six weeks after birth	15(16.7%)	84(31.3%)
	Four weeks after birth	10(11.1%)	5 (1.8%)
	Don’t know	35(38.9%)	7 (2.5%)
How many times should a child vaccinated	One	17(18.5%)	5(1.8%)
	Two	19 (20.4%)	12(4.3%)
	Three	5(5.6%)	25(9.2%)
	Four	13(13%)	132(49.1%)
	Five	5(5.8%)	87(32.5%)
	Don’t know	31(33.9%)	8 (3.1%)
Age of vaccination completion	14 weeks age	15(16.7%)	16 (6.1%)
	at nine months	22(24.1%)	226(84%)
	Others	8(9.3%)	18(6.7%)
	Don’t know	45(50%)	9(3.1%)

### 3.4. Child related factors for vaccination dropout

The main reasons for vaccination dropout were child sickness 5 (5.6%) cases; forgot appointments 15 (16.7%) cases; of mothers/ care givers, 28 (31.5%) cases don’t know continuation phases of vaccination. From the total 73 (81.5%) of cases and 157 (58.5%) of controls fear vaccine side effect to complete vaccination (Table 4).

**Table 4:** Factors for vaccination dropout among children age 12–23 months in north Gondar, northwest Ethiopia, 2019 (n = 359).

Variables	Category	Vaccination status	
		Cases	Controls
Sex of child	Male	41(45.3%)	132(49%)
	Female	49(54.7%)	137(51%)
Reason of vaccination dropout	Child was sick during schedule	9(9.6%)	20(7.4%)
	Forgot appointment	11(12%)	14(5.3%)
	I don’t know to continue the 2^nd^,3^rd^, and/or 4rth doses	28(31.5%)	30(11%)
	Others	42(46.9%)	205(76.3%)
Fear of vaccine’s side effect	Yes	73(81.5%)	157 (58.5%)
	No	17(18.5%)	112(41.7%)

### 3.5. Institutional related factors of vaccination dropout

The results of institutional related factors showed that the majority of health facilities exist nearby to 53(59.3%) of cases were health posts, and to 246 (91.4%) of controls were health centers. The major means of transportation of mothers/caregivers to health facilities were on feet for 84(93.4%) of cases and 258 (95.9%) of controls.

This study showed that 8(8.5%) of cases and 132 (49%) of controls lived within less than 15 minutes distance from a health facility. Twenty two (24.1%) of cases and 76 (28.2%) of controls missed vaccination opportunity for different reasons. This study also showed that home visit was done by HEW/HW for 33 (37%) of cases and 213 (79.1%) of controls (Table 5).

**Table 5:** Institutional related factors of children on determinants of vaccination dropout among children age 12–23 months in north Gondar, northwest Ethiopia, 2019 (n = 359).

Variables	Category	Vaccination status	
		Cases	Controls
Presence of nearby health facility	Yes	53(59.3%)	246(91.4%)
	No	37(40.7%)	23(8.6%)
Type of health facility exist nearby	Health center	30(33.8%)	155(57.7%)
	Health post	53(58.8%)	99(36.7%)
	Private clinic	7(7.9%)	15(5.6%)
Means of transport	On walk	84(93.4%)	258(95.9%)
	Transportations	6(6.6%)	11(4.1%)
Time to reach health facility	Less than 15 minutes	8(8.5%)	132(49%)
	15–30 minutes	22(24%)	53(19.6%)
	30–60 minutes	5(5.6%)	12(4.4%)
	More than one hour	49(55.6%)	56(21%)
	I don’t know	6(6.3%)	16(6%)
Counseling on vaccination	Yes	72(79.6)	167(62%)
	No	18(20.4%)	122(38%)
Missing vaccination opportunity	Yes	13(24.1%)	76(28.2%)
	No	41(78.9%)	193(71.8%)
If missed why(yes)	No provider at that day	6 (46.1%)	35(46%)
	Vaccination stock out	3 (23.2%)	28(36.8%)
	Vaccination day postponed	4 (30.7)	13(17.2)
Home visit by HEW/HW	Yes	56(62.2%)	186(69.3%)
	No	34(37.8%)	83(30.7%)
If yes how many times	Every one month	11(19.6%)	93(34.4%)
	Every two month	7(12.5%)	103(38.4%)
	Every quarter	15(26.8%)	50(18.4%)
	Others	23(41.1%)	23(8.8%)

### 3.6. Determinants of vaccination dropout

On binary logistic regression analysis eleven variables at p-value <0.25 were fitted for multivariable logistic regression model. Counseling on child vaccination, age of mothers/care givers, knowledge on the benefits of vaccination, heard about vaccination risk/benefit, fear of vaccine side effects, marital status, postnatal care, TT vaccination, fear of vaccine side effect, sex of child, way of life were significantly associated with vaccination status. In the multivariable logistic regression analysis only four of them were found significant and were risk factors of defaulting from completion of child immunization after adjusted for confounding, at 95% CI with p-value < 0.05.

The result showed that respondents who had not got counseling from health workers during vaccination session and other maternal service were seven times more likely to dropout child vaccination as compared to those counseled (AOR = 7.2, 95% CI: (2.93–17.5). Respondents who had fear of vaccine side effects were three times more likely to default child vaccination as compared with respondents who had no fear of vaccine side effects (AOR = 3.5, 95% CI: (1.56–8.12). Mothers/care givers who were not attended PNC were 3.05 times more likely to dropout child vaccination than their counterparts (AOR = 3.6, 95% CI: (1.52–8.39). Comparing mothers of children who had vaccinated TT at least once during pregnancy, child born from mothers who were not vaccinated, were two times more likely to dropout child vaccination than those who were got TT vaccination (AOR = 2.4; 95% CI;1.03–5.35) (Table 6).

**Table 6:** Bivariable and multivariable logistic regression analysis on determinants of vaccination dropout among children age 12–23 months in north Gondar, northwest Ethiopia, 2019 (n = 359).

Variables	Category	Vaccination status		Odds Ratio at 95% CI	
		Cases	Controls	Crude OR	Adjusted OR
Marital status	Married	65(72.2%)	249(92.6%)	3.87(1.18–12.66)	3.18(0.74–13.60)
	Widowed	12(13.1%)	10(3.7%)	0.86(0.18–4.13)	0.40(0.06–2.89)
	Divorced	13(14.7%)	10(3.7%)	1	1
PNC attended	Yes	48(53.7%)	101(37.4%)	1	1
	No	42(46.3%)	168(62.6%)	1.90(1.28–7.96)	3.05 (2.42–5.84)*
Counseling on vaccination	Yes	72(79.6)	167(62%)	1	1
	No	18(20.4%)	102(38%)	2.44(3.05–13.26))	7.17(2.93–17.5)**
Fear of vaccine side effects	Yes	57(63%)	83(30.7%)	3.87(2.01–7.32)*	3.56(1.56–8.12)*
	No	33(37%)	186(69.3%)	1	1
Home visit by HEW/HW	Yes	73(81.5%)	157(58.5%)	0.32(0.15–0.68)	2.23(0.86–5.78)
	No	17(18.5%)	112(41.7%)	1	1
Heard about vaccination	Yes	75(83.3%)	259(96.3%)	1	1
	No	15(16.7%)	10(3.7%)	0.19(0.07–0.56)*	0.32(0.07–1.45)
TT vaccine received	Yes	32(35.2%)	137(50.9%)	1	1
	No	58(64.8%)	132(49.1%)	1.91(1.01–3.62)*	2.35(1.03–5.35)*

1 = reference category

* = significant at p<0.05

** = significant at p<0.001 in multivariable logistic regression

## 4. Discussion

Immunization is one of the most powerful and cost-effective public health interventions. It prevents debilitating illness and disability, and saves millions of lives every year. Vaccines have the power not only to save life, but also to giving children a chance to grow up healthy and improve their life prospects. Though the immunization coverage is improving, the program is challenged with vaccination dropouts. However, Immunization will become more effective if the child can receive the full course of recommended vaccination doses [2].

This study provides the analysis of determinants of vaccination dropout among 90 cases and 269 controls. Counseling on vaccination was one of the variables significantly associated with vaccination status of children. Children born from mothers who had not get counseling about vaccination from health workers during vaccination session and other maternal service were seven times more likely to dropout vaccination as compared with those mothers who were counseled. This finding was similar with the studies conducted in Yirgalem town, south Ethiopia [20] and urban areas of Kenya [21]. But it was different from the study done in Arbegona districts of southern Ethiopia [22]. The possible explanation for this difference might be due to inadequate information provision about the importance of child vaccination completion during receiving maternal and child health service from health personnel and socio-cultural differences of the study [4].

In this study, those mothers/care givers who had fear of vaccine side effects were 3 times more likely to default immunization as compared with those who had no fear. This result was supported by findings of studies conducted in Tigray, Oromia and Somali regions of Ethiopia [23–25] respectively. This may be due to lack of health education and awareness creation efforts; and poor home visit rounds by health workers for reducing mothers fear about vaccine side effect.

The likelihood of defaulting from completion of child vaccination was about three times more likely among mothers/care givers who were not attended PNC after birth as compared to those attended at least once. This study was consistent with study conducted in Kenya that revealed failure to attend PNC was considered as an obstacle to complete vaccination [21]. The possible explanation of this findings may be loss of chances to access advice on benefits of child vaccination that causes mothers/care givers to default from child immunization. Tetanus toxoid (TT) vaccination status was significantly associated with the fully completion of child vaccination. Mothers who were not vaccinated at least one dose of Tetanus Toxoid during their ANC follow ups were two times more likely to dropout vaccination as compared with mothers who had received tetanus toxoid vaccination during their ANC follow up. This study was consistent with study conducted in Debramarkos [23], Lay Armachiho [26], Ambo [27], Hosanna town, south Ethiopia [4], Jigjiga [28] and Bangladesh [29] that revealed mothers who hadn’t received TT vaccination during pregnancy were less likely to complete vaccination than who received tetanus toxoid vaccination. The possible explanation could be mothers were not visited health facilities for tetanus toxoid immunization and might have no exposure to information about the importance of completing vaccination.

## 5. Limitation of the study

The study had some limitations which included recall bias where mother who was not possess EPI card might forget the vaccination status of their children and misclassification. Cultural factors and perceptions were not studied during this study.

## 6. Conclusion

Counseling on vaccination, fear of vaccine side effects, status of PNC attendance and tetanus toxoid vaccination during ANC were found risk factors of vaccination dropout among children aged 12–23 months. Therefore, there is a need to advice those mothers’ who visit at health facilities for maternal health service on child vaccination completion in any opportunity. Better to strength home visit of the catchment households so that tracing vaccination dropout and delivering health education about the importance of child vaccination completion. An innovative approach like “mobile health strategy” and outreach services need to be strengthened to decrease vaccination dropout among the community.

## Abbreviation

AOR: Adjusted Odds Ratio
ANC: Ante Natal Care
CI: Confidence Interval
COR: Crude Odds Ratio
DPT: Diphtheria, Pertussis and Tetanus
EDHS: Demographic and Health Survey
EPI: Expanded Program on immunization
GVAP: Global Vaccine Action Plan
HepB: Hepatitis B
Hib: Hemophilus influenza type b
OPV: Oral Polio Vaccine
PCV: Pneumococcal conjugated Vaccine
PHCU: Primary Health Care Unit
PNC: Post Natal Care
REC: Reach every community
